# The role of plasma microseminoprotein-beta in prostate cancer: an observational nested case–control and Mendelian randomization study in the European prospective investigation into cancer and nutrition

**DOI:** 10.1093/annonc/mdz121

**Published:** 2019-04-08

**Authors:** K Smith Byrne, P N Appleby, T J Key, M V Holmes, G K Fensom, A Agudo, E Ardanaz, H Boeing, H B Bueno-de-Mesquita, M D Chirlaque, R Kaaks, N Larrañaga, D Palli, A Perez-Cornago, J R Quirós, F Ricceri, M J Sánchez, G Tagliabue, K K Tsilidis, R Tumino, R T Fortner, P Ferrari, PRACTICAL Consortium, E Riboli, H Lilja, R C Travis

**Affiliations:** 1 Cancer Epidemiology Unit; 2 Medical Research Council Population Health Research Unit, University of Oxford, Oxford; 3 Clinical Trial Service Unit & Epidemiological Studies Unit (CTSU), Oxford; 4 National Institute for Health Research Oxford Biomedical Research Centre, Oxford University Hospital, Oxford; 5 Medical Research Council Integrative Epidemiology Unit, University of Bristol, Bristol, UK; 6 Unit of Nutrition and Cancer, Catalan Institute of Oncology-IDIBELL, Barcelona; 7 CIBER of Epidemiology and Public Health (CIBERESP), Madrid; 8 Navarra Public Health Institute, Pamplona; 9 Navarra Institute for Health Research (IdiSNA), Pamplona, Spain; 10 Department of Epidemiology, German Institute of Human Nutrition (DIfE), Potsdam-Rehbrücke, Germany; 11 Department for Determinants of Chronic Diseases (DCD), National Institute for Public Health and the Environment (RIVM), Bilthoven, The Netherlands; 12 Department of Epidemiology and Biostatistics, Imperial College London, London, UK; 13 Department of Social & Preventive Medicine, Faculty of Medicine, University of Malaya, Kuala Lumpur, Malaysia; 14 Department of Epidemiology, IMIB-Arrixaca, Murcia; 15 Department of Health and Social Sciences, University of Murcia, Murcia, Spain; 16 Division of Cancer Epidemiology, German Cancer Research Center (DKFZ), Heidelberg, Germany; 17 Public Health Division of Gipuzkoa, Regional Government of the Basque Country, Vitoria-Gasteiz, Spain; 18 Cancer Risk Factors and Life-style Epidemiology Unit, Institute for Cancer Research, Prevention and Clinical Network (ISPRO), Florence, Italy; 19 Public Health Directorate, Asturias, Spain; 20 Unit of Epidemiology, Regional Health Service Azienda Sanitaria Locale Torino 3 (ASL TO3), Grugliasco; 21 Unit of Cancer Epidemiology, Department of Medical Sciences, University of Turin, Turin, Italy; 22 Escuela Andaluza de Salud Pública, Instituto de Investigación Biosanitaria ibs.GRANADA, Hospitales Universitarios de Granada/Universidad de Granada, Granada, Spain; 23 Department of Preventative and Predictive Medicine, Fondazione IRCCS Istituto Nazionale dei Tumori, Milan, Italy; 24 Department of Hygiene and Epidemiology, University of Ioannina School of Medicine, Ioannina, Greece; 25 Cancer Registry and Histopathology Unit, “Civic M.P. Arezzo” Hospital, Ragusa, Italy; 26 Nutritional Methodology and Biostatistics Group, International Agency for Research on Cancer (IARC/WHO), Lyon, France; 27 Nuffield Department of Surgical Sciences, University of Oxford, Oxford, UK; 28 Department of Laboratory Medicine, Memorial Sloan Kettering Cancer Center, New York, USA; 29 Department of Surgery, Memorial Sloan Kettering Cancer Center, New York, USA; 30 Department of Medicine, Memorial Sloan Kettering Cancer Center, New York, USA; 31 Department of Translational Medicine, Lund University, Malmö, Sweden

**Keywords:** prostate cancer, microseminoprotein-beta, prostate-specific antigen, prospective study, EPIC cohort, Mendelian randomization

## Abstract

**Background:**

Microseminoprotein-beta (MSP), a protein secreted by the prostate epithelium, may have a protective role in the development of prostate cancer. The only previous prospective study found a 2% reduced prostate cancer risk per unit increase in MSP. This work investigates the association of MSP with prostate cancer risk using observational and Mendelian randomization (MR) methods.

**Patients and methods:**

A nested case–control study was conducted with the European Prospective Investigation into Cancer and Nutrition (EPIC) with 1871 cases and 1871 matched controls. Conditional logistic regression analysis was used to investigate the association of pre-diagnostic circulating MSP with risk of incident prostate cancer overall and by tumour subtype. EPIC-derived estimates were combined with published data to calculate an MR estimate using two-sample inverse-variance method.

**Results:**

Plasma MSP concentrations were inversely associated with prostate cancer risk after adjusting for total prostate-specific antigen concentration [odds ratio (OR) highest versus lowest fourth of MSP = 0.65, 95% confidence interval (CI) 0.51–0.84, *P*trend = 0.001]. No heterogeneity in this association was observed by tumour stage or histological grade. Plasma MSP concentrations were 66% lower in rs10993994 TT compared with CC homozygotes (per allele difference in MSP: 6.09 ng/ml, 95% CI 5.56–6.61, *r*^2^=0.42). MR analyses supported a potentially causal protective association of MSP with prostate cancer risk (OR per 1 ng/ml increase in MSP for MR: 0.96, 95% CI 0.95–0.97 versus EPIC observational: 0.98, 95% CI 0.97–0.99). Limitations include lack of complete tumour subtype information and more complete information on the biological function of MSP.

**Conclusions:**

In this large prospective European study and using MR analyses, men with high circulating MSP concentration have a lower risk of prostate cancer. MSP may play a causally protective role in prostate cancer.

**Key Message**

Using observational data and Mendelian randomization we found a protective association of a protein produced by the prostate, microseminoprotein-beta, with prostate cancer risk.

## Introduction 

Microseminoprotein-beta (MSP) is a protein secreted by the prostate epithelium into the seminal fluid [1]. In the only previous prospective study, the Multiethnic Cohort (MEC) [2], a 1 ng/ml increase in circulating MSP concentration was associated with a 2% decrease in prostate cancer risk. MSP concentrations, in both blood and semen samples from healthy males, are ∼60% higher among CC homozygotes versus TT homozygotes for rs10993994 (*r*^2^ = 0.38 and 0.23, respectively), located 57 base-pairs upstream in the 5′ promoter region of the *MSMB* gene [3], which encodes the protein MSP. Furthermore, a genome-wide association study (GWAS) has found carriers of the T allele to have an elevated prostate cancer risk (57% higher for TT versus CC) [2, 4].

This prospective study investigated whether circulating MSP concentrations were associated with prostate cancer risk in the European Prospective Investigation into Cancer and Nutrition (EPIC). We then investigated the association of rs10993994 with circulating concentrations of MSP in EPIC and used this genetic variant as an instrument for MSP to assess its potential causal role through Mendelian randomization (MR) analyses by combining EPIC-derived estimates with published data from the Prostate Cancer Association Group to Investigate Cancer Associated Alterations in the Genome (PRACTICAL) consortium [5].

## Methods

### Study population

Totally, 137 000 men participating in EPIC provided blood samples at recruitment between 1992 and 2000 [6]. Lifestyle questionnaires, anthropometric data, and food questionnaires were collected at recruitment. All participants provided written informed consent. Approval for the study was obtained from the ethical review boards of the participating institutions and the International Agency for Research on Cancer (IARC). The current study uses data from Germany, Greece, Italy, the Netherlands, Spain and the UK.

### Follow-up

Cancer incidence was identified through record linkage to regional or national registries in most countries (see supplementary methods, available at *Annals of Oncology* online). Follow-up procedures continued to prostate cancer diagnosis or last follow-up completed (31 December 2007 to 14 June 2010).

Cases were men who were diagnosed with incident prostate cancer (International Classification of Diseases 10th revision code C61 [7]) after blood collection and before the end of follow-up. An incidence density sampling protocol was used to select control participants at random from the cohort of men who were alive and free of cancer (excluding non-melanoma skin cancer) at the time of diagnosis of the index case and who matched on study centre, length of follow-up, age at blood collection, time of blood collection and duration of fasting at blood collection. These analyses included 1871 cases with 1871 matched controls. Information on tumour stage and grade at diagnosis was available for 1263 (67.5%) and 1554 (85.1%) of cases, respectively (see supplementary methods, available at *Annals of Oncology* online).

### Assessment of analytes

Immunoassay measurements for prostate-specific antigen (PSA) [8] and MSP [9, 10] were conducted on the AutoDelfia^®^ 1235 automatic immunoassay system in Dr Lilja’s laboratory at the Wallenberg Research Laboratories, Department of Translational Medicine, Lund University, Skåne University Hospital, Malmö, Sweden (see supplementary methods, available at *Annals of Oncology* online).

### Statistical analysis

Analyte concentrations below limits of detection were set to half the lowest concentration (PSA, *N *=* *7), and concentrations above the upper limits were set to the highest value for that analyte (MSP, *N *=* *82; PSA, *N *=* *65). Pearson’s *χ*^2^ tests and paired *t*-tests were conducted between matched case**–**control sets for anthropometric and lifestyle characteristics. Analysis of variance was used to assess differences in analyte concentrations in controls by strata of selected characteristics, country and study phase (matched case**–**control sets were identified after each of three rounds of follow-up and end point data centralisation in EPIC conducted in approximately 2004, 2008 and 2010, and samples from each phase were assayed together). Log transformations were applied to analyte concentrations and results are presented as geometric means adjusted for age at blood collection, body mass index (BMI), recruitment centre and laboratory batch.

Conditional logistic regression models were used to examine the association of MSP with prostate cancer, conditioned on the matching factors and adjusted for BMI, age at blood collection and further adjusted for fourth of PSA concentration (additional adjustment was shown to not materially alter the results, see supplementary Table S1, available at *Annals of Oncology* online). These analyses were repeated in subgroups according to study phase, time between blood collection and diagnosis, age at blood collection, age at diagnosis, prostate tumour stage and histological grade. Additional unconditional analyses stratified by median PSA concentration and smoking status were adjusted for age, BMI, fourth of PSA concentration and matching factors. Linear trend was tested using a pseudo-continuous variable equal to medians of the fourths of MSP concentration. For subgroup analyses, likelihood ratio tests were used to test for heterogeneity.rs10993994 genotype data were available for a subset of 1068 EPIC cases and 1186 EPIC controls from the iCOGS [11], OncoArray [12] and Breast and Prostate Cancer Cohort Consortium (BPC3) [13] genotyping projects. Logistic regression models were used to investigate the association of rs10993994 with prostate cancer.

We investigated the potential causal role of MSP in prostate cancer risk using MR analyses. A summary estimate of the association of rs10993994 with prostate cancer was taken from the iCOGS genotyping project in the international consortium PRACTICAL with 25 000 cases from 32 studies [5, 11], and from EPIC prostate cancer cases and controls genotyped in the OncoArray [12] and BPC3 studies [13]. Summary estimates for the association of rs10993994 with MSP were calculated using these EPIC data [12, 13]. We used the MR-Base platform to do a phenome-wide association scan for rs10993994 with 850 traits to check for pleiotropy [14], and also checked the NHGR-EBI catalogue of published GWAS [15]. Two-sample MR estimates were calculated separately using summary estimates for each of PRACTICAL (iCOGS) [5] and EPIC-derived rs10993994-prostate cancer risk estimates with the EPIC-derived rs10993994-MSP estimate, which were then combined using the inverse-variance weighted method. To address possible confounding by PSA, we conducted sensitivity analyses using the summary association of rs10993994 with residuals from a linear regression of log total PSA on MSP, also calculated within EPIC.

All statistical tests are two-sided and were conducted using STATA software version 14 (StataCorp LP, College Station, TX).

## Results

Data from 1871 cases and 1871 matched controls were included in the analyses. The median age at blood collection was 58 years, and, for cases, the median time between blood collection and diagnosis was 8.3 years. No significant differences were observed in selected baseline characteristics between cases and controls (Table 1).

**Table 1.** Characteristics of control participants and prostate cancer patients

Characteristics^e^	Controls (*n *=* *1871)	Cases (*n *=* *1871)	*P* ^a^
Age at blood collection (years)^b^	58.3 (6.9)	58.3 (6.9)	0.5
Weight (kg)^b^	80.2 (11.6)	80.2 (11.5)	0.5
Height (cm)^b^	172.9 (7.2)	172.5 (7.1)	0.9
BMI (kg/m^2^)^b^	26.9 (3.5)	27.1 (3.5)	0.2
Smoking status, *n* (%)			
Never	578 (31.5)	621 (34.5)	
Previous	826 (45.1)	792 (43.9)	
Current	431 (23.4)	389 (21.6)	0.1
Alcohol, *n* (%)			
<8	657 (34.8)	682 (36.4)	
8–15	368 (19.9)	363 (19.4)	
16–39	542 (28.9)	491 (26.2)	
>40	304 (16.3)	335 (17.9)	0.3
Physical activity, *n* (%)			
Inactive	277 (15.1)	268 (14.9)	
Moderately inactive	533 (29.1)	525 (29.4)	
Active	1025 (55.9)	996 (55.7)	0.9
Marital status, *n* (%)			
Married/cohabitating	1377 (89.7)	1333 (88.6)	
Not married/cohabitating	160 (10.4)	172 (11.4)	0.3
Educational attainment, *n* (%)			
Primary/none	687 (38.3)	668 (38.3)	
Secondary	633 (35.4)	596 (34.1)	
Degree	471 (26.3)	482 (27.6)	0.6
Geometric mean analyte concentration at blood collection			
MSP (ng/ml) (95% CI)	12.8 (12.5–13.2)	12.9 (12.6–13.2)	0.7
MSP adjusted for PSA (ng/ml) (95% CI)	12.9 (12.6–13.3)	12.8 (12.5–13.1)	0.5
PSA (ng/ml) (95% CI)	0.8 (0.8–0.9)	2.4 (2.3–2.5)	<0.0001
Time to diagnosis, *n* (%)			
<2 years		81 (4.4)	
2 to <4 years		111 (5.9)	
4 to <6 years		244 (13.1)	
6 to <8 years		375 (20.2)	
8 to <10 years		1049 (56.4)	
Year of diagnosis, median (range)		2004 (1994–2009)	
Age at diagnosis (years) (SD)		66.9 (6.9)	
Tumour stage			
TNM-code^c^			
Tumour			
T1		176 (19.4)	
T2		529 (58.4)	
T3		183 (20.2)	
T4		18 (1.9)	
Nodes			
N0		609 (92.3)	
N1		46 (6.9)	
N2		4 (0.1)	
N3		1 (0.01)	
Metastases			
M0		522 (94.7)	
M1		29 (5.3)	
EPIC stage information^c^			
Localized		778 (82.4)	
Metastatic		205 (21.7)	
Tumour grade			
Gleason grade^d^			
≤6		452 (55.7)	
7		218 (28.6)	
≥8		120 (15.7)	
EPIC grade information^d^			
Well differentiated		139 (16.6)	
Moderately differentiated		503 (60.1)	
Poorly differentiated		191 (22.8)	
Undifferentiated		4 (0.1)	
PSA (ng/ml) at diagnosis			
<3		20 (3.6)	
≥3 and <10		335 (59.8)	
≥10 and <50		187 (33.4)	
≥50		18 (3.2)	

a *P*-values are from analysis of variance models where characteristics are continuous and *χ*^2^ test where characteristics are categorical.

b Geometric means are presented with standard deviation.

c TNM-code and EPIC stage are not mutually exclusive as some individuals had information for both.

d Gleason grade and EPIC grade are not mutually exclusive as some individuals had information for both.

e Numbers may not sum to total due to missing values.

BMI, body mass index; MSP, microseminoprotein-beta; CI, confidence interval; PSA, prostate-specific antigen; SD, standard deviation; EPIC, European Prospective Investigation into Cancer and Nutrition.

Mean MSP concentration (ng/ml) at blood collection did not differ significantly between cases and controls (Table 1). Mean PSA concentration (ng/ml) measured at blood collection was about threefold higher in cases than controls [adjusted geometric means = 2.4, 95% confidence interval (CI) 2.3–2.5 and 0.8, 0.8–0.9 respectively, *P *<* *0.0001].

MSP concentration in controls was higher in men older at blood collection, not married, with normal/low BMI or low-alcohol intake, and who had higher educational attainment (*P *<* *0.05 for all). Compared with never smokers, men who smoked more than 15 cigarettes per day had 30% higher MSP concentrations (*P*trend < 0.0001). PSA concentration was positively associated with age at blood collection and educational attainment, and negatively associated with greater BMI and diabetes (Table 2). MSP and PSA concentrations were positively correlated in both cases and controls (partial correlations *r* = 0.3 and 0.2, respectively, *P *<* *0.0001).

**Table 2.** Adjusted geometric mean MSP and PSA concentration (ng/ml) in controls by selected characteristics

Factor and subset	MSP (ng/ml)			PSA (ng/ml)		
	*N*	Mean (95% CI)^a^	*P*-difference/linear trend^b^	*N*	Mean (95% CI)^a^	*P*-difference/linear trend^b^
Age at blood collection (years)						
<50	195	11.7 (10.8–12.6)		195	0.6 (0.5–0.6)	
50–55	339	12.3 (11.6–13.1)		339	0.7 (0.6–0.7)	
55–59	503	12.2 (11.7–12.9)		503	0.8 (0.7–0.9)	
60–64	549	12.7 (12.1–13.3)		549	0.9 (0.9–1.0)	
65–69	169	15.4 (14.2–16.8)		169	1.3 (1.1–1.4)	
>70	116	16.8 (15.2–18.6)	<0.0001/<0.0001	116	1.6 (1.3–1.8)	<0.0001/<0.0001
Time of blood collection (h)						
00:00–09:59	305	13.0 (12.1–13.9)		306	0.9 (0.8–1.0)	
10:00–12:59	267	13.6 (12.6–14.6)		267	0.8 (0.7–0.9)	
13:00–23:59	1176	12.4 (12.0–12.9)	0.1	1176	0.8 (0.8–0.9)	0.03
Marital status						
Married/cohabitating	1377	12.9 (12.5–13.2)		1377	0.8 (0.8–0.9)	
Not married/cohabitating	160	14.3 (13.1–15.6)	0.01	160	0.9 (0.8–1.0)	0.9
Educational attainment						
Primary/none	687	12.3 (11.8–12.8)		687	0.8 (0.7–0.8)	
Secondary	633	13.2 (12.7–13.8)		633	0.9 (0.8–0.9)	
Degree	471	12.8 (12.2–13.5)	0.02	471	0.9 (0.8–0.9)	0.02
Body mass index (kg/m^2^)						
16–24	542	13.7 (13.1–14.3)		542	0.9 (0.8–0.9)	
25–29	1003	12.8 (12.3–13.2)		1003	0.8 (0.8–0.9)	
>30	317	11.7 (10.9–12.4)	0.0007/0.001	317	0.7 (0.7–0.8)	0.02/<0.001
Smoking status						
Never	578	11.9 (11.4–12.4)		578	0.9 (0.8–0.9)	
Previous	826	12.1 (11.7–12.6)		826	0.8 (0.8–0.9)	
Current (<15 cigarettes)	181	15.8 (14.6–17.1)		181	0.9 (0.8–0.9)	
Current (≥15 cigarettes)	154	17.0 (15.6–18.6)	<0.0001/<0.0001	154	0.8 (0.7–0.9)	0.9
Usual alcohol consumption (g/day)						
<8	657	13.6 (13.0–14.2)		657	0.9 (0.8–0.9)	
8–15	368	13.0 (12.3–13.8)		368	0.9 (0.8–0.9)	
16–39	542	12.3 (11.8–12.9)		542	0.8 (0.8–0.9)	
>40	304	11.9 (11.2–12.7)	0.002/<0.0001	304	0.8 (0.7–0.9)	0.3
Diabetic						
No	1760	12.9 (12.5–13.2)		1760	0.9 (0.8–0.9)	
Yes	97	12.1 (10.8–13.5)	0.5	97	0.7 (0.6–0.8)	0.02

a All means adjusted for age at blood collection, body mass index, recruitment centre and batch; adjustment not made for age at blood collection excluded for strata of age at blood collection, nor body mass index for strata of body mass index.

b *P-*values are from analysis of variance and, where significant difference was observed and dose-dependent relationship implied, from test for linear trend.

MSP, microseminoprotein-beta; PSA, prostate-specific antigen; CI, confidence interval.

MSP concentration was not associated with prostate cancer risk after adjustment for age at blood collection and BMI [odds ratio (OR) for highest versus lowest fourth = 0.98, 95% CI 0.82–1.19, *P*trend = 0.9)]. However, after adjustment for PSA, MSP concentration was associated with prostate cancer risk (OR = 0.65, 95% CI 0.51–0.84, *P*trend = 0.001) (Table 3). There was some evidence of heterogeneity in the association by time to diagnosis (with a stronger association in men diagnosed within 8.5 years of baseline, *P*heterogeneity = 0.009), age at diagnosis (*P*heterogeneity = 0.03); (supplementary Table S2, available at *Annals of Oncology* online) and recruitment country (*P*heterogeneity = 0.02; supplementary Table S3, available at *Annals of Oncology* online). There was no significant heterogeneity of risk by smoking status (*P*heterogeneity = 0.6; supplementary Table S2, available at *Annals of Oncology* online).

**Table 3.** Multi-variable adjusted odds ratios (95% CI) for prostate cancer by fourth of plasma MSP concentration, subdivided by selected factors

		Fourth of MSP concentration (ng/ml)					
		1	2	3	4	*P* for trend^a^	*P* for heterogeneity of trends^b^
Overall	Cases/controls, *n*	508/468	402/464	458/468	501/469		
	Median MSP (ng/ml) (range)	7 (1–9)	12 (9–13)	16 (13–18)	29 (18–90)		
	Basic OR (95% CI)^c^	1 (reference)	0.82 (0.68–0.99)	0.91 (0.76–1.09)	0.98 (0.82–1.19)	0.9	
	Adjusted OR (95% CI)^d^	1 (reference)	0.84 (0.65–1.09)	0.75 (0.58–0.97)	0.65 (0.51–0.84)	0.001	
Stage^e^							
Localised (*n*=886)	Cases/controls, *n*	243/229	194/221	214/209	235/225		
	Adjusted OR (95% CI)^d^	1 (reference)	0.86 (0.57–1.28)	0.77 (0.52–1.15)	0.64 (0.44–0.92)	0.02	
Advanced (*n*=377)	Cases/controls, *n*	110/95	87/81	91/109	89/92		
	Adjusted OR (95% CI)^d^	1 (reference)	0.79 (0.44–1.43)	0.45 (0.25–0.79)	0.45 (0.24–0.82)	0.002	0.2
Grade^e^ (Gleason ≥8 cut-off)							
Low-intermediate (*n*=1357)	Cases/controls, *n*	384/349	281/338	346/354	345/315		
	Adjusted OR (95% CI)^d^	1 (reference)	0.83 (0.59–1.15)	0.76 (0.56–1.02)	0.63 (0.46–0.86)	0.004	
High (*n*=197)	Cases/controls, *n*	53/49	44/48	36/38	63/62		
	Adjusted OR (95% CI)^d^	1 (reference)	0.86 (0.42–1.76)	0.68 (0.32–1.46)	0.73 (0.39–1.42)	0.3	0.7
Death from prostate cancer (*n*=169)							
	Cases/controls, *n*	43/45	38/42	46/40	42/42		
	Basic OR (95% CI)^c^	1 (reference)	1.06 (0.59–1.94)	0.89 (0.47–1.67)	0.98 (0.54–1.77)	0.8	
	Adjusted OR (95% CI)^d^	1 (reference)	0.84 (0.38–1.86)	0.59 (0.27–1.30)	0.40 (0.18–0.89)	0.02	

a Test for trend was obtained by replacing the categorical variable with a continuous variable equal to the median concentration within each fourth of plasma MSP concentration.

b Test for heterogeneity in the trends.

c Estimates are from logistic regression conditioned on the matching variables: centre, age at blood collection, follow-up time, fasting status and time of day at blood collection, with adjustment for age and body mass index (continuous).

d Additional to model ‘b’, adjustment was made for body mass index (fourths), and total PSA (fourths).

e Tumour stage information was available for 1263 (67.5%) cases: 886 cases were clinically localized (defined as tumour–node–metastasis staging score of T1–T2 and N0/Nx and M0/Mx, or stage coded in the recruitment centre as localized); 377 were clinically advanced (T3–T4 and/or N1–N3 and/or M1, or stage coded in the recruitment centre as metastatic). Tumour grade information at diagnosis was available for 1554 cases (85.1%): 1357 were low-intermediate grade (defined as Gleason score <8, or grade coded as well, moderately or poorly differentiated) and 197 were high-grade (Gleason score ≥8, or grade coded as undifferentiated).

CI, confidence interval; MSP, microseminoprotein-beta; OR, odds ratio.

The association of MSP with prostate cancer did not differ by tumour stage or grade, or age at blood collection (all *P*heterogeneity ≥ 0.05; Table 3 and supplementary Table S2, available at *Annals of Oncology* online). Results were not materially altered and no significant heterogeneity was observed with high grade defined as Gleason score ≥7 (supplementary Table S2, available at *Annals of Oncology* online). MSP was associated with risk of death from prostate cancer (OR = 0.40, 95% CI 0.18–0.89, with adjustment for age, BMI and PSA; Table 3).

PSA concentration was strongly and positively associated with risk for prostate cancer, both with and without adjustment for MSP concentration (OR = 45.2, 95% CI 29.7–68.7, with adjustment for age, BMI and MSP; supplementary Table S4, available at *Annals of Oncology* online).

In a subset of 1068 cases and 1186 controls with rs10993994 genotype data there was a 6.09 ng/ml (95% CI 5.56–6.61) per allele difference in MSP concentration, with highest concentrations observed for CC homozygotes. rs10993994 explained 42% of the variability of MSP. In controls, there was a 0.22 ng/ml (95% CI 0.09–0.35) per allele difference in PSA concentrations, with highest concentrations observed for TT homozygotes (supplementary Table S5, available at *Annals of Oncology* online). In this EPIC dataset, rs10993994 genotype was significantly associated with prostate cancer (OR CC versus TT = 0.73, 95% CI 0.57–0.93, *P*trend = 0.006) (supplementary Table S6, available at *Annals of Oncology* online).

After correction for multiple testing, no significant association of rs10993994 genotype was observed with potential confounders beyond PSA concentrations in controls (supplementary Table S7, available at *Annals of Oncology* online). PheWAS using published data [14, 15], showed that besides prostate cancer risk, rs10993994 is associated only with the prostate cancer biomarkers PSA and prostate cancer antigen 3 (PCA3) at the genome-wide significance level. An inverse-variance weighted MR showed a one unit increase in circulating MSP concentrations (ng/ml) is associated with a 4% reduction in prostate cancer risk (OR = 0.96, 95% CI 0.95–0.97) (Table 4), and was not altered after adjustment for PSA (supplementary Table S8, available at *Annals of Oncology* online).

**Table 4.** Odds ratio for prostate cancer risk per unit increase in MSP (ng/ml) for IV estimates and MR results using inverse-variance method

Study	OR per unit increase in MSP (ng/ml) (95% CI)
PRACTICAL for incident cancer	0.96 (0.95–0.98)
EPIC (excluding PRACTICAL) for incident cancer	0.97 (0.95–0.99)
All pooled	0.96 (0.95–0.97)

MSP, microseminoprotein-beta; IV, instrumental variable; MR, Mendelian randomization; OR, odds ratio; CI, confidence interval; PRACTICAL, Prostate Cancer Association Group to Investigate Cancer Associated Alterations in the Genome; EPIC, European Prospective Investigation into Cancer and Nutrition.

## Discussion

In this large prospective study, we found a lower prostate cancer risk in men with higher circulating concentrations of MSP after adjustment for circulating PSA concentrations. MSP is a protein in the immunoglobulin-binding factor family primarily secreted by epithelial cells, which may have a role in tumour suppression[16] and pathogen defence [17]. These findings are in agreement with the only other published prospective investigation [2], which found an inverse association between circulating MSP concentration and prostate cancer; in the MEC study, MSP concentration was inversely associated with prostate cancer risk before and after adjustment for PSA, though the association was much stronger after adjustment, as is to be expected due to the strong positive association of PSA concentration with risk and the moderate positive association of PSA with MSP. In accordance with previous findings [2], we found no evidence that the association of MSP with risk differed by tumour stage or grade, although small numbers of cases in subgroups may have limited power to evaluate heterogeneity. We found some modest evidence for heterogeneity by country and age at diagnosis, but the results are difficult to interpret due to small numbers in subgroups and multiple statistical tests.

Short follow-up time (3.8 years) and thus reverse causality was previously suggested in MEC as a possible explanation for the observed association. The present study has more than double the average follow-up (8.3 years), and while we found some observational evidence that the inverse association between MSP and prostate cancer is stronger for men diagnosed closer to blood collection, the apparent differences by time to diagnosis may be at least in part due to differences in the case mix, with cases diagnosed closer to baseline being more likely to be younger at diagnosis. Furthermore, our MR findings suggest that reverse causality is unlikely to explain the overall relationship, with genetic variation in MSP affecting lifetime levels of MSP.

MSP is also secreted at lower levels by epithelial cells in the tracheobronchial tree [18, 19]. Smoking has been associated with a 2.5-fold increase in expression of MSP in the airway epithelium when compared with non-smokers [20]. Therefore, some variation in MSP concentrations may be due to smoking-induced secretory cell hyperplasia in the respiratory tract. To our knowledge, we are the only study to report higher levels of MSP among current smokers compared with non-smokers. We found no strong evidence of heterogeneity in the MSP association by smoking status but more data are needed to examine this and particularly to assess the association in non-smokers in whom any potential masking effect of smoking on circulating MSP is not present.

The strength of the current MR result stems from the use of rs10993994 as an instrumental variable; rs10993994 lies in the promotor region of the *MSMB* region, the locus that encodes MSP, and rs10993994 is strongly associated with circulating MSP concentrations and prostate cancer [2, 5]. In general, the use of variants in the *cis*-acting protein-encoding locus is one of the most robust scenarios of MR [21] and a recent review of MSP function [22] suggest the rs10993994 genetic association is specific to MSP. An association of rs10993994 has been observed with concentrations of prostate cancer markers PSA and PCA3 in prostate cancer controls [15], and it remains possible that PSA may confound these results. However, given that the associations of rs10993994 with PSA and PCA3 levels are observed only in controls and that MR results were materially robust to adjustment for PSA concentration, the association of rs10993994 with PSA (and PCA3) may arise from collider bias. Such collider bias [23], which induces the association of rs10993994 with PSA and PCA3 when stratifying on prostate cancer disease status, should not invalidate the results of the MR analysis (which is not stratified on disease status). Additionally, for the biological role of PSA to confound these findings, PSA would have to be causal to prostate cancer development for which there is little evidence.

### Conclusion

Using observational data from a prospective nested case**–**control study and MR, this study supports a possible protective role of MSP in the development of prostate cancer. Experimental studies are needed to elucidate the mechanisms through which MSP may influence prostate cancer development. If shown to be true from randomized clinical trials, therapies that raise MSP levels may provide novel opportunities for the treatment and prevention of prostate cancer.

## Supplementary Material

mdz121_Supplementary_Data

## References

[1] Lilja H, Abrahamsson PA. Three predominant proteins secreted by the human prostate gland. Prostate 1988; 12(1): 29–38. 3347596 10.1002/pros.2990120105

[2] Haiman CA, Stram DO, Vickers AJ et al Levels of beta-microseminoprotein in blood and risk of prostate cancer in multiple populations. J Natl Cancer Inst 2013; 105(3): 237–243. 23213189 10.1093/jnci/djs486 PMC3565627

[3] Xu X, Valtonen AC, Sävblom C et al Polymorphisms at the microseminoprotein-β locus associated with physiologic variation in β-microseminoprotein and prostate-specific antigen levels. Cancer Epidemiol Biomarkers Prev 2010; 19: 2035–2042. 20696662 10.1158/1055-9965.EPI-10-0431 PMC2946372

[4] Eeles RA, Kote-Jarai Z, Giles GG et al Multiple newly identified loci associated with prostate cancer susceptibility. Nat Genet 2008; 40(3): 316–321. 18264097 10.1038/ng.90

[5] Kote-Jarai Z, Easton DF, Stanford JL et al Multiple novel prostate cancer predisposition loci confirmed by an international study: the PRACTICAL Consortium. Cancer Epidemiol Biomarkers Prev 2008; 17(8): 2052–2061. 18708398 10.1158/1055-9965.EPI-08-0317 PMC2776652

[6] Riboli E, Hunt K, Slimani N et al European Prospective Investigation into Cancer and Nutrition (EPIC): study populations and data collection. Public Health Nutr 2002; 5(6b): 1113–1124. 12639222 10.1079/PHN2002394

[7] Worl Health Organization. ICD-10. International Statistical Classification of Diseases and Related Health Problems, 10th edition. Geneva, Switzerland: World Health Organization (WHO) 1992.

[8] Mitrunen K, Pettersson K, Piironen T et al Dual-label one-step immunoassay for simultaneous measurement of free and total prostate-specific antigen concentrations and ratios in serum. Clin Chem 1995; 41(8 Pt 1): 1115–1120. 7543033

[9] Sjöblom L, Saramäki O, Annala M et al Microseminoprotein-beta expression in different stages of prostate cancer. PLoS One 2016; 11(3): e0150241. 26939004 10.1371/journal.pone.0150241 PMC4777373

[10] Valtonen‐André C, Sävblom C, Fernlund P et al Beta‐microseminoprotein in serum correlates with the levels in seminal plasma of young, healthy males. J Androl 2008; 29: 330–337. 18222915 10.2164/jandrol.107.003616

[11] Eeles RA, Al Olama AA, Benlloch S et al Identification of 23 new prostate cancer susceptibility loci using the iCOGS custom genotyping array. Nat Genet 2013; 45(4): 385–391. 23535732 10.1038/ng.2560 PMC3832790

[12] Amos CI, Dennis J, Wang Z et al The OncoArray Consortium: a network for understanding the genetic architecture of common cancers. Cancer Epidemiol Biomarkers Prev 2017; 26(1): 126–135. 27697780 10.1158/1055-9965.EPI-16-0106 PMC5224974

[13] Hunter D, Riboli E, Haiman C et al A candidate gene approach to searching for low-penetrance breast and prostate cancer genes. Nat Rev Cancer 2005; 5: 977–985. 16341085 10.1038/nrc1754

[14] Hemani G, Zheng J, Wade KH et al MR-Base: a platform for systematic causal inference across the phenome using billions of genetic associations. bioRxiv 2016 [Preprint]; 078972.

[15] Buniello A, MacArthur JAL, Cerezo M et al The NHGRI-EBI GWAS Catalog of published genome-wide association studies, targeted arrays and summary statistics 2019. Nucleic Acids Research 2019; 47(Database issue): D1005-D1012. 10.1093/nar/gky1120 PMC6323933 30445434

[16] Garde S, Sheth A, Porter AT, Pienta KJ. Effect of prostatic inhibin peptide (PIP) on prostate cancer cell growth in vitro and in vivo. Prostate 1993; 22(3): 225–233. 8488155 10.1002/pros.2990220305

[17] Hägerwall AME, Rydengård V, Fernlund P et al β-Microseminoprotein endows post coital seminal plasma with potent candidacidal activity by a calcium-and pH-dependent mechanism. PLoS Pathog 2012; 8: e1002625. 22496651 10.1371/journal.ppat.1002625 PMC3320615

[18] Weiber H, Andersson C, Murne A et al Beta microseminoprotein is not a prostate-specific protein. Its identification in mucous glands and secretions. Am J Pathol 1990; 137: 593–603. 2205099 PMC1877516

[19] Ulvsback M, Lindstrom C, Weiber H et al Molecular cloning of a small prostate protein, known as beta-microsemenoprotein, PSP94 or beta-inhibin, and demonstration of transcripts in non-genital tissues. Biochem Biophys Res Commun 1989; 164(3): 1310–1315. 2590204 10.1016/0006-291x(89)91812-3

[20] Tilley A, Staudt M, Fuller J et al Smoking-induced up-regulation of microseminoprotein beta gene expression in the human airway. Am J Respir Crit Care Med 2012; 185: A6052.

[21] Swerdlow DI, Kuchenbaecker KB, Shah S et al Selecting instruments for Mendelian randomization in the wake of genome-wide association studies. Int J Epidemiol 2016; 45(5): 1600–1616. 27342221 10.1093/ije/dyw088 PMC5100611

[22] Sutcliffe S, De Marzo AM, Sfanos KS, Laurence M. *MSMB* variation and prostate cancer risk: clues towards a possible fungal etiology. Prostate 2014; 74(6): 569–578. 24464504 10.1002/pros.22778 PMC4037912

[23] Day FR, Loh P-R, Scott RA et al A robust example of collider bias in a genetic association study. Am J Hum Genet 2016; 98(2): 392. 26849114 10.1016/j.ajhg.2015.12.019 PMC4746366

