# Supplementary material for: The role of plasma microseminoprotein-beta in prostate cancer: an observational nested case–control and Mendelian randomization study in the European prospective investigation into cancer and nutrition
Source: Ann Oncol. 2019 Apr 8;30(6):983–9. doi: 10.1093/annonc/mdz121 (PMC6594452; doi:10.1093/annonc/mdz121)
Supplement: mdz121_Supplementary_Data [file mdz121_supplementary_data.zip › mdz121-Suppl_data/Supplementary methods.docx]

**Supplementary methods**

**Follow-up**

For Germany and Greece, combined health insurance records, regional health departments, municipality registries, hospital or physician-based cancer and pathology records, or mail or phone call-based follow-up were used.

Cases were men who were diagnosed with incident prostate cancer (International Classification of Diseases 10th revision code C61[1]) after blood collection and before the end of follow-up. An incidence density sampling protocol was used to select control participants at random from the cohort of men who were alive and free of cancer (excluding non-melanoma skin cancer) at the time of diagnosis of the index case and who matched on study centre, length of follow-up, age at blood collection (±6 months), time of blood collection (±1 hour) and duration of fasting at blood collection (<3, 3-6, >6 hours). These analyses included 1,871 cases with 1,871 matched controls.

886 cases were clinically localized (tumor-node-metastasis staging score of T1-T2 and N0/Nx and M0/Mx, or stage coded in the recruitment centre as localized); 377 were clinically advanced (T3-T4 and/or N1-N3 and/or M1, or stage coded in the recruitment centre as metastatic). Tumor grade information at diagnosis was available for 1,554 cases (85.1%): 1,357 were low-intermediate grade (Gleason score<8, or grade coded as well, moderately, or poorly differentiated) and 197 were high-grade (Gleason score≥8, or grade coded as undifferentiated).

**Assessment of Analytes**

PSA was measured using the dual-label DELFIA Prostatus® total PSA-Assay (Perkin-Elmer, Turku, Finland) [2] calibrated against the WHO 96/670 (PSA-WHO) standard, with blinding of laboratory staff to case-control status. Production and purification of the polyclonal rabbit anti-MSP antibody, protocols for biotinylation and Europium labeling of the anti-MSP antibody, and performance of the MSP-immunoassay were performed as previously reported [3, 4].

Quality control samples were inserted into each assay batch and analysed in duplicate. The intra- and inter-assay coefficients of variation ranged from 5-15% for MSP and 7-14% for PSA. The concordance between measurements of microseminoprotein-beta (MSP) and prostate-specific antigen (PSA) concentration in serum and citrated plasma samples was assessed (*N*=25 for serum and plasma, respectively). A high concordance was observed between concentrations measured in serum and in plasma (r=0.98). Additionally, the temporal reproducibility of analyte concentrations was assessed between samples drawn at five year intervals from 49 and 40 individuals for MSP and PSA, respectively. There were no significant differences between serum concentrations of MSP (*p*=0.4) or PSA (*p*=0.8) drawn at five year intervals. Given the wider availability of plasma samples than serum samples for the EPIC cohort, all assays for the nested case-control study were performed using plasma.

**Members of the PRACTICAL Consortium**

Brian E. Henderson, Mariana C. Stern, Alison Thwaites, Michelle Guy, Ian Whitmore, Angela Morgan, Cyril Fisher, Steve Hazel, Naomi Livni, Margaret Cook, Laura Fachal, Stephanie Weinstein, Laura E. Beane Freeman, Robert N. Hoover, Mitchell J. Machiela, Artitaya Lophatananon, Brian D. Carter, Phyllis Goodman, Leire Moya, Srilakshmi Srinivasan, Mary-Anne Kedda, Trina Yeadon, Allison Eckert, Martin Eklund, Carin Cavalli-Bjoerkman, Alison M. Dunning, Csilla Sipeky, Niclas Hakansson, Rebecca Elliott, Hardeep Ranu, Edward Giovannucci, Constance Turman, David J. Hunter, Olivier Cussenot, Torben Falck Orntoft, Athene Lane, Sarah J. Lewis, Michael Davis, Tim J. Key, Paul Brown, Girish S. Kulkarni, Alexandre R. Zlotta, Neil E. Fleshner, Antonio Finelli, Xueying Mao, Jacek Marzec, Robert J. MacInnis, Roger Milne, John L. Hopper, Miguel Aguado, Mariona Bustamante, Gemma Castaño-Vinyals, Esther Gracia-Lavedan, Lluís Cecchini, Meir Stampfer, Jing Ma, Thomas A. Sellers, Milan S. Geybels, Hyun Park, Babu Zachariah, Suzanne Kolb, Dominika Wokolorczyk, Jan Lubinski, Wojciech Kluzniak, Sune F. Nielsen, Maren Weisher, Katarina Cuk, Walther Vogel, Manuel Luedeke, Christopher J. Logothetis, Paula Paulo, Marta Cardoso, Sofia Maia, Maria P. Silva, Linda Steele, Yuan Chun Ding, Gert De Meerleer, Sofie De Langhe, Hubert Thierens, Jasmine Lim, Meng H. Tan, Aik T. Ong, Daniel W. Lin, Darina Kachakova, Atanaska Mitkova, Vanio Mitev, Matthew Parliament, Guido Jenster, Christopher Bangma, F. H. Schroder, Thérèse Truong, Yves Akoli Koudou, Agnieszka Michael, Andrzej Kierzek, Ami Karlsson, Michael Broms, Huihai Wu, Claire Aukim-Hastie, Lori Tillmans, Shaun Riska, Shannon K. McDonnell, David Dearnaley, Amanda Spurdle, Robert Gardiner, Vanessa Hayes, Lisa Butler, Renea Taylor, Melissa Papargiris, Pamela Saunders, Paula Kujala, Kirsi Talala, Kimmo Taari, Søren Bentzen, Belynda Hicks, Aurelie Vogt, Amy Hutchinson, Angela Cox, Anne George, Ants Toi, Andrew Evans, Theodorus H. van der Kwast, Takashi Imai, Shiro Saito, Shan-Chao Zhao, Guoping Ren, Yangling Zhang, Yongwei Yu, Yudong Wu, Ji Wu, Bo Zhou, John Pedersen, Ramón Lobato-Busto, José Manuel Ruiz-Dominguez, Lourdes Mengual, Antonio Alcaraz, Julio Pow-Sang, Kathleen Herkommer, Aleksandrina Vlahova, Tihomir Dikov, Svetlana Christova, Angel Carracedo, Brigitte Tretarre, Xavier Rebillard, Claire Mulot, Jan Adolfsson, Par Stattin, Jan-Erik Johansson, Richard M. Martin, Ian M. Thompson Jr., Suzanne Chambers, Joanne Aitken, Lisa Horvath, Anne-Maree Haynes, Wayne Tilley, Gail Risbridger, Markus Aly, Tobias Nordström, Paul Pharoah, Teuvo L. J Tammela, Teemu Murtola, Anssi Auvinen, Neil Burnet, Gill Barnett, Gerald Andriole, Aleksandra Klim, Bettina F. Drake, Michael Borre, Sarah Kerns, Harry Ostrer, Hong-Wei Zhang, Guangwen Cao, Ji Lin, Jin Ling, Meiling Li, Ninghan Feng, Jie Li, Weiyang He, Xin Guo, Zan Sun, Guomin Wang, Jianming Guo, Melissa C. Southey, Liesel M FitzGerald, Gemma Marsden, Antonio Gómez-Caamaño, Ana Carballo, Paula Peleteiro, Patricia Calvo, Robert Szulkin, Javier Llorca, Trinidad Dierssen-Sotos, Ines Gomez-Acebo, Hui-Yi Lin, Elaine A. Ostrander, Rasmus Bisbjerg, Peter Klarskov, Martin Andreas Røder, Peter Iversen, Bernd Holleczek, Christa Stegmaier, Thomas Schnoeller, Philipp Bohnert, Esther M. John, Piet Ost, Soo-Hwang Teo, Marija Gamulin, Tomislav Kulis, Zeljko Kastelan, Chavdar Slavov, Elenko Popov, Thomas Van den Broeck, Steven Joniau, Samantha Larkin, Jose Esteban Castelao, Maria Elena Martinez, Ron H. N. van Schaik, Jianfeng Xu, Sara Lindstrӧm, Elio Riboli, Clare Berry, Afshan Siddiq, Federico Canzian, Laurence N. Kolonel, Loic Le Marchand, Matthew Freedman, Sylvie Cenee & Marie Sanchez

**References**

1. Organization WH. ICD-10. International statistical classification of diseases and related health problems (10th edition). Geneva, SR: World Health Organization (WHO) 1992.

2. Mitrunen K, Pettersson K, Piironen T et al. Dual-label one-step immunoassay for simultaneous measurement of free and total prostate-specific antigen concentrations and ratios in serum. Clinical Chemistry 1995; 41: 1115-1120.

3. Sjöblom L, Saramäki O, Annala M et al. Microseminoprotein-Beta Expression in Different Stages of Prostate Cancer. PloS one 2016; 11: e0150241.

4. Valtonen‐André C, Sävblom C, Fernlund P et al. Beta‐Microseminoprotein in Serum Correlates With the Levels in Seminal Plasma of Young, Healthy Males. Journal of Andrology 2008; 29: 330-337.

5. Kote-Jarai Z, Easton DF, Stanford JL et al. Multiple novel prostate cancer predisposition loci confirmed by an international study: the PRACTICAL Consortium. Cancer Epidemiol Biomarkers Prev 2008; 17: 2052-2061.

6. Cox DG, Blanché H, Pearce CL et al. A comprehensive analysis of the androgen receptor gene and risk of breast cancer: results from the National Cancer Institute Breast and Prostate Cancer Cohort Consortium (BPC3). Breast Cancer Research 2006; 8: R54.

7. Amos CI, Dennis J, Wang Z et al. The OncoArray Consortium: a Network for Understanding the Genetic Architecture of Common Cancers. Cancer Epidemiology and Prevention Biomarkers 2017; 26: 126-135.
