# Supplementary material for: The role of plasma microseminoprotein-beta in prostate cancer: an observational nested case–control and Mendelian randomization study in the European prospective investigation into cancer and nutrition
Source: Ann Oncol. 2019 Apr 8;30(6):983–9. doi: 10.1093/annonc/mdz121 (PMC6594452; doi:10.1093/annonc/mdz121)
Supplement: mdz121_Supplementary_Data [file mdz121_supplementary_data.zip › mdz121-Suppl_data/Supplementary Table S1.docx]

| **Supplementary Table S1**. The impact of adjustment factors on the odds ratio for the association of MSP with prostate cancer risk^a^ | | |
| --- | --- | --- |
| Model | OR (95% CI)^d^ | *P* for trend^e^ |
| Basic model^b^ | 0.65 (0.51 to 0.84) | 0.003 |
|  |  |  |
| Adjusted models |  |  |
| Basic + smoking status | 0.65 (0.50 to 0.85) | 0.004 |
| Basic + alcohol consumption | 0.65 (0.51 to 0.84) | 0.003 |
| Basic + marital status | 0.66 (0.51 to 0.85) | 0.003 |
| Basic + total physical activity | 0.65 (0.51 to 0.84) | 0.003 |
| Basic + educational attainment | 0.66 (0.51 to 0.85) | 0.003 |
|  |  |  |
| Fully adjusted model^c^ | 0.66 (0.51 to 0.86) | 0.005 |
| ^a^ MSP = microseminoprotein-beta; OR = odds ratio; CI = confidence interval. | | |
| ^b^ Basic model adjusted for total PSA ( fourths), age at blood collection, body mass index (fourths) and recruitment centre. | | |
| ^c^ Fully adjusted model is the basic model with additional adjustment for smoking status, alcohol consumption, marital status, total physical activity, and educational attainment. | | |
| ^d^ Odds ratio for comparison between highest and lowest quartile of MSP. | | |
| ^e^ Test for trend obtained by replacing the categorical variable with a continuous variable equal to the median concentration within each fourth of plasma MSP concentration. | | |
