# Supplementary material for: The role of plasma microseminoprotein-beta in prostate cancer: an observational nested case–control and Mendelian randomization study in the European prospective investigation into cancer and nutrition
Source: Ann Oncol. 2019 Apr 8;30(6):983–9. doi: 10.1093/annonc/mdz121 (PMC6594452; doi:10.1093/annonc/mdz121)
Supplement: mdz121_Supplementary_Data [file mdz121_supplementary_data.zip › mdz121-Suppl_data/Supplementary Table S2.docx]

| **Supplementary Table S2.**  Multi-variable adjusted odds ratio (95% CI) for prostate cancer by fourth of MSP concentration, subdivided by selected factors^a^ | | | | | | | | | | |
| --- | --- | --- | --- | --- | --- | --- | --- | --- | --- | --- |
| Factor | |  | Fourth of MSP concentration (ng/ml) | | | |  | |  | |
|  |  |  | 1 | 2 | 3 | 4 | | *P* for trend^c^ | | *P* for heterogeneity of trends^d^ |
| **Study Phase** | |  |  |  |  |  | |  | |  |
| Phase 1 | | Cases/controls, *n* | 72/88 | 53/67 | 61/47 | 60/44 | |  | |  |
|  | | Adjusted OR (95% CI)^b^ | 1 (reference) | 0.62 (0.22 to 1.75) | 0.96 (0.38 to 2.39) | 0.52 (0.19 to 1.36) | | 0.2 | |  |
| Phase 2 | | Cases/controls, *n* | 130/111 | 121/135 | 162/173 | 233/227 | |  | |  |
|  | | Adjusted OR (95% CI)^b^ | 1 (reference) | 0.77 (0.45 to 1.32) | 0.59 (0.37 to 0.98) | 0.59 (0.38 to 0.94) | | 0.03 | |  |
| Phase 3 | | Cases/controls, *n* | 306/269 | 228/262 | 235/248 | 208/198 | |  | |  |
|  | | Adjusted OR (95% CI)^b^ | 1 (reference) | 0.85 (0.61 to 1.17) | 0.77 (0.56 to 1.07) | 0.65 (0.46 to 0.91) | | 0.01 | | 0.9 |
| **Time between blood collection and diagnosis** | | |  |  |  |  | |  | |  |
| < 8.5 years | | Cases/controls, *n* | 211/234 | 224/192 | 229/238 | 264/264 | |  | |  |
|  | | Adjusted OR (95% CI)^b^ | 1 (reference) | 0.62 (0.39 to 0.98) | 0.53 (0.35 to 0.82) | 0.40 (0.26 to 0.61) | | 0.001 | |  |
| ≥ 8.5 years | | Cases/controls, *n* | 257/273 | 235/210 | 237/215 | 201/232 | |  | |  |
|  | | Adjusted OR (95% CI)^b^ | 1 (reference) | 0.94 (0.67 to 1.30) | 0.80 (0.58 to 1.11) | 0.86 (0.62 to 1.20) | | 0.3 | | 0.009 |
| **Age at blood collection** | | |  |  |  |  | |  | |  |
| < 60 years | | Cases/controls, *n* | 314/279 | 242/287 | 260/270 | 214/194 | |  | |  |
|  | | Adjusted OR (95% CI)^b^ | 1 (reference) | 0.79 (0.56 to 1.13) | 0.65 (0.46 to 0.91) | 0.68 (0.46 to 0.99) | | 0.03 | |  |
| ≥ 60 years | | Cases/controls, *n* | 189/184 | 155/171 | 193/194 | 281/269 | |  | |  |
|  | | Adjusted OR (95% CI)^b^ | 1 (reference) | 0.89 (0.58 to 1.35) | 0.89 (0.59 to 1.32) | 0.63 (0.44 to 0.90) | | 0.007 | | 0.5 |
| **Age at diagnosis** | |  |  |  |  |  | |  | |  |
| < 65 years | | Cases/controls, *n* | 218/194 | 177/193 | 183/191 | 146/146 | |  | |  |
|  | | Adjusted OR (95% CI)^b^ | 1 (reference) | 0.77 (0.47 to 1.25) | 0.45 (0.27 to 0.74) | 0.45 (0.26 to 0.78) | | 0.001 | |  |
| ≥ 65 years | | Cases/controls, *n* | 290/274 | 225/271 | 275/277 | 355/323 | |  | |  |
|  | | Adjusted OR (95% CI)^b^ | 1 (reference) | 0.84 (0.61 to 1.16) | 0.89 (0.67 to 1.21) | 0.74 (0.55 to 0.99) | | 0.05 | | 0.03 |
| **Stage** | |  |  |  |  |  | |  | |  |
| Localised | | Cases/controls, *n* | 243/229 | 194/221 | 214/209 | 235/225 | |  | |  |
|  | | Adjusted OR (95% CI)^b^ | 1 (reference) | 0.86 (0.57 to 1.28) | 0.77 (0.52 to 1.15) | 0.64 (0.44 to 0.92) | | 0.02 | |  |
| Advanced | | Cases/controls, *n* | 110/95 | 87/81 | 91/109 | 89/92 | |  | |  |
|  | | Adjusted OR (95% CI)^b^ | 1 (reference) | 0.79 (0.44 to 1.43) | 0.45 (0.25 to 0.79) | 0.45 (0.24 to 0.82) | | 0.002 | | 0.2 |
|  | |  |  |  |  |  | |  | |  |
|  | |  |  |  |  |  | |  | |  |
|  | |  |  |  |  |  | |  | |  |
| **Supplementary Table S2.** Continued | | |  |  |  |  | |  | |  |
| **Grade**  (Gleason ≥7 cut -off) | |  |  |  |  |  | |  | |  |
| Low to intermediate | | Cases/controls, *n* | 263/249 | 190/234 | 239/246 | 235/196 | |  | |  |
|  | | Adjusted OR (95% CI)^b^ | 1 (reference) | 0.94 (0.59 to 1.51) | 0.99 (0.65 to 1.50) | 0.84 (0.54 to 1.29) | | 0.2 | |  |
| High | | Cases/controls, *n* | 173/146 | 133/149 | 139/144 | 172/178 | |  | |  |
|  | | Adjusted OR (95% CI)^b^ | 1 (reference) | 0.81 (0.39 to 1.18) | 0.72 (0.47 to 1.13) | 0.55 (0.36 to 0.83) | | 0.004 | | 0.2 |
| **Strata of smoking status** |  | |  |  |  |  | |  | |  |
| Never/previous | Cases/controls, *n* | | 422/394 | 330/374 | 331/351 | 329/293 | |  | |  |
|  | Adjusted OR (95% CI)^e^ | | 1 (reference) | 0.84 (0.65 to 1.08) | 0.68 (0.53 to 0.88) | 0.66 (0.51 to 0.87) | | 0.001 | |  |
| Current | Cases/controls, *n* | | 71/72 | 61/80 | 110/110 | 147/168 | |  | |  |
|  | Adjusted OR (95% CI)^e^ | | 1 (reference) | 0.92 (0.52 to 1.64) | 0.88 (0.53 to 1.48) | 0.77 (0.46 to 1.26) | | 0.3 | | 0.6 |
| **By median PSA (0.78 ng/ml)** | Adjusted OR (95% CI)^b^ | | 1 (reference) | 0.92 (0.52 to 1.64) | 0.88 (0.53 to 1.48) | 0.77 (0.46 to 1.26) | | 0.3 | |  |
| Below median PSA | Cases/controls, *n* | | 48/259 | 44/288 | 40/214 | 28/170 | |  | |  |
|  | Adjusted OR (95% CI)^e^ | | 1 (reference) | 0.84 (0.53 to 1.33) | 0.96 (0.59 to 1.55) | 0.91 (0.54 to 1.55) | | 0.8 | |  |
| Above median PSA | Cases/controls, *n* | | 460/210 | 359/176 | 418/254 | 473/299 | |  | |  |
|  | Adjusted OR (95% CI)^e^ | | 1 (reference) | 0.92 (0.71 to 1.19) | 0.71 (0.56 to 0.90) | 0.55 (0.43 to 0.69) | | < 0.0001 | | 0.02 |
| ^a^ MSP = microseminoprotein-beta; CI = confidence interval; OR = odds ratio; PSA = prostate-specific antigen. | | | | | | | | | | |
| ^b^ Estimates are from logistic regression conditioned on the matching variables: centre, age at blood collection, follow up time, fasting status and time of day of blood collection, with adjustment for age and body mass index (fourths) and total PSA (fourths). | | | | | | | | | | |
| ^c^ Test for trend obtained by replacing the categorical variable with a continuous variable equal to the median concentration within each fourth of plasma MSP concentration. | | | | | | | | | | |
| ^d^ Test for heterogeneity of the trends. | | | | | | | | | | |
| ^e^ Conditional matching was broken and a logistic regression adjusting for matching factors was performed. | | | | | | | | | | |
