# Supplementary material for: The role of plasma microseminoprotein-beta in prostate cancer: an observational nested case–control and Mendelian randomization study in the European prospective investigation into cancer and nutrition
Source: Ann Oncol. 2019 Apr 8;30(6):983–9. doi: 10.1093/annonc/mdz121 (PMC6594452; doi:10.1093/annonc/mdz121)
Supplement: mdz121_Supplementary_Data [file mdz121_supplementary_data.zip › mdz121-Suppl_data/Supplementary Table S3.docx]

| **Supplementary Table S3.** Multi-variable adjusted odds ratio (95% CI) for prostate cancer by fourth of plasma MSP concentration, subdivided by country^a^ | | | | | | | | |
| --- | --- | --- | --- | --- | --- | --- | --- | --- |
|  |  | Fourth of MSP concentration (ng/ml) | | | |  | |  |
|  |  | 1 | 2 | 3 | 4 | *P* for trend^c^ | *P* for heterogeneity of trends^d^ | |
| **Country** |  |  |  |  |  |  |  | |
| Germany | Cases/controls, *n* | 232/200 | 146/187 | 163/160 | 146/140 |  |  | |
|  | Adjusted OR (95% CI)^b^ | 1 (reference) | 0.60 (0.38 to 0.93) | 0.51 (0.32 to 0.81) | 0.46 (0.29 to 0.72) | < 0.0001 |  | |
| Greece | Cases/controls, *n* | 18/22 | 14/19 | 23/20 | 20/14 |  |  | |
|  | Adjusted OR (95% CI)^b^ | 1 (reference) | 1.02 (0.21 to 4.95) | 0.72 (0.18 to 2.90) | 1.12 (0.27 to 4.66) | 0.9 |  | |
| Italy | Cases/controls, *n* | 81/74 | 66/76 | 61/70 | 64/52 |  |  | |
|  | Adjusted OR (95% CI)^b^ | 1 (reference) | 1.21 (0.56 to 2.58) | 0.75 (0.38 to 1.49) | 1.05 (0.51 to 2.19) | 0.8 |  | |
| The Netherlands | Cases/controls, *n* | 29/23 | 23/26 | 32/28 | 24/31 |  |  | |
|  | Adjusted OR (95% CI)^b^ | 1 (reference) | 0.75 (0.25 to 2.25) | 1.09 (0.39 to 3.03) | 0.27 (0.08 to 0.94) | 0.07 |  | |
| Spain | Cases/controls, *n* | 66/77 | 64/62 | 70/75 | 71/57 |  |  | |
|  | Adjusted OR (95% CI)^b^ | 1 (reference) | 1.17 (0.64 to 2.16) | 1.19 (0.66 to 2.19) | 1.58 (0.86 to 2.92) | 0.2 |  | |
| The United Kingdom | Cases/controls, *n* | 82/72 | 89/93 | 109/115 | 175/175 |  |  | |
|  | Adjusted OR (95% CI)^b^ | 1 (reference) | 0.91 (0.49 to 1.69) | 0.68 (0.38 to 1.22) | 0.49 (0.29 to 0.87) | 0.004 | 0.02 | |
| ^a^ CI = confidence interval; MSP = microseminoprotein-beta; OR = odds ratio; PSA = prostate-specific antigen. | | | | | | | | |
| ^b^ Estimates are from logistic regression conditioned on the matching variables: centre, age at blood collection, follow up time, fasting status and time of day of blood collection, with adjustment for age, body mass index (fourths) and total PSA (fourths). | | | | | | | | |
| **^c^** Test for trend obtained by replacing the categorical variable with a continuous variable equal to the median concentration within each fourth of plasma MSP concentration. | | | | | | | | |
| ^d^ Test for heterogeneity of the trends. | | | | | | | | |
