# Supplementary material for: The role of plasma microseminoprotein-beta in prostate cancer: an observational nested case–control and Mendelian randomization study in the European prospective investigation into cancer and nutrition
Source: Ann Oncol. 2019 Apr 8;30(6):983–9. doi: 10.1093/annonc/mdz121 (PMC6594452; doi:10.1093/annonc/mdz121)
Supplement: mdz121_Supplementary_Data [file mdz121_supplementary_data.zip › mdz121-Suppl_data/Supplementary Table S4.docx]

| **Supplementary Table S4.** Odds ratios (95% CI) for prostate cancer by fourth of plasma PSA concentration ^a^ | | | | | | | | |
| --- | --- | --- | --- | --- | --- | --- | --- | --- |
|  |  | Fourth of PSA concentration | | | |  | |  |
|  |  | 1 | 2 | 3 | 4 | *P* for trend^d^ |  |  |
| **Overall** | Cases/controls, *n* | 43/447 | 109/452 | 353/462 | 1309/453 |  |  |  |
|  | Basic OR (95% CI)^b^ | 1 (reference) | 2.40 (1.56 to 3.67) | 8.00 (5.36 to 11.94) | 42.93 (28.31 to 65.10) | <0.001 |  |  |
|  | Adjusted OR (95% CI)^c^ | 1 (reference) | 2.43 (1.58 to 3.73) | 8.10 (5.42 to 12.10) | 45.17 (29.70 to 68.69) | <0.001 |  |  |

^a^ CI = confidence interval; MSP = microseminoprotein-beta; OR = odds ratio; PSA = prostate-specific antigen.

^b^ Estimates are from logistic regression conditioned on the matching variables: centre, age at blood collection, follow up time, fasting status and time of day at blood collection, with adjustment for age and body mass index (continuous).

^c^ Additional to model ‘b’, adjustment was made for body mass index (fourths), and MSP concentration (fourths).

^d^  Test for trend was obtained by replacing the categorical variable with a continuous variable equal to the median concentration within each fourth of plasma PSA concentration.
