# Supplementary material for: The role of plasma microseminoprotein-beta in prostate cancer: an observational nested case–control and Mendelian randomization study in the European prospective investigation into cancer and nutrition
Source: Ann Oncol. 2019 Apr 8;30(6):983–9. doi: 10.1093/annonc/mdz121 (PMC6594452; doi:10.1093/annonc/mdz121)
Supplement: mdz121_Supplementary_Data [file mdz121_supplementary_data.zip › mdz121-Suppl_data/Supplementary Table S5.docx]

| **Supplementary Table S5.** Multi-variable adjusted geometric mean concentration of MSP and total PSA concentration at recruitment by rs10993994 genotype and case/control status for prostate cancer^a^ | | | | | | | | | | | | | | | |
| --- | --- | --- | --- | --- | --- | --- | --- | --- | --- | --- | --- | --- | --- | --- | --- |
|  | rs10993994 genotype | | | | | | | | |  | |  |  |  |  |
|  | CC | |  | | CT | |  | TT | |  |  | | | | |
| Case/control status | *N* | Adjusted mean^b^  (95% CI) | | *N* | | Adjusted mean^b^  (95% CI) | *N* | | Adjusted mean^b^  (95% CI) |  | *P* for genotype differences | | | | |
| **MSP (ng/ml)** |  |  |  | | |  |  | |  |  |  | | | | |
| Control | 398 | 18.1 (17.4 to 18.9) | 571 | | | 12.9 (12.5 to 13.4) | 208 | | 5.9 (5.6 to 6.3) |  | <0.0001 | | | | |
| Case | 298 | 19.2 (18.3 to 20.1) | 545 | | | 13.2 (12.7 to 13.7) | 215 | | 6.3 (5.9 to 6.7) |  | <0.0001 | | | | |
| **Total PSA (ng/ml)** |  |  |  | | |  |  | |  |  |  | | | | |
| Control | 398 | 0.7 (0.7 to 0.8) | 571 | | | 0.8 (0.7 to 0.9) | 208 | | 0.9 (0.7 to 1.1) |  | 0.0004 | | | | |
| Case | 298 | 2.3 (2.1 to 2.5) | 545 | | | 2.4 (2.3 to 2.6) | 215 | | 2.3 (2.1 to 2.6) |  | 0.6 | | | | |
| ^a^ MSP = microseminoprotein-beta; PSA = prostate-specific antigen; CI = confidence interval. | | | | | | | | | | | | | | | |
| ^b^ Adjusted for age, body mass index, recruitment centre and batch. | | | | | | | | | | | | | | | |
|  | | | | | | | | | | | | | | | |
