# Supplementary material for: The role of plasma microseminoprotein-beta in prostate cancer: an observational nested case–control and Mendelian randomization study in the European prospective investigation into cancer and nutrition
Source: Ann Oncol. 2019 Apr 8;30(6):983–9. doi: 10.1093/annonc/mdz121 (PMC6594452; doi:10.1093/annonc/mdz121)
Supplement: mdz121_Supplementary_Data [file mdz121_supplementary_data.zip › mdz121-Suppl_data/Supplementary Table S6.docx]

| **Supplementary Table S6.** Odds ratio (95% CI) for prostate cancer by rs10993994^a^ | | | |
| --- | --- | --- | --- |
| rs10993994 | Case/control | OR(95% CI) | *P* for trend |
| TT | 215/208 | 1(Reference) |  |
| CT | 552/578 | 0.92 (0.74-1.16) |  |
| CC | 301/400 | 0.73 (0.57 to 0.93) | 0.006 |
| ^a^ CI = confidence interval; OR = odds ratio. | | | |
