# Supplementary material for: The role of plasma microseminoprotein-beta in prostate cancer: an observational nested case–control and Mendelian randomization study in the European prospective investigation into cancer and nutrition
Source: Ann Oncol. 2019 Apr 8;30(6):983–9. doi: 10.1093/annonc/mdz121 (PMC6594452; doi:10.1093/annonc/mdz121)
Supplement: mdz121_Supplementary_Data [file mdz121_supplementary_data.zip › mdz121-Suppl_data/Supplementary Table S7.docx]

| **Supplementary Table S7.** Characteristics of participants by rs10993994 genotype^a^ | | | | |
| --- | --- | --- | --- | --- |
|  | rs10993994 genotype | | |  |
| Characteristic | CC | CT | TT | *P*^b^ |
| *N* | 701 | 1,130 | 423 |  |
| Age at blood collection, years^c^ | 57.94 (57.46 to 58.42) | 58 (57.62 to 58.38) | 57.91 (57.3 to 58.52) | 0.9 |
| Weight, kg^c^ | 80.36 (79.5 to 81.22) | 80.14 (79.49 to 80.79) | 80.08 (78.98 to 81.18) | 0.9 |
| Height, cm^c^ | 172.34 (171.82 to 172.86) | 172.28 (171.88 to 172.68) | 172.38 (171.67 to 173.09) | 0.9 |
| BMI, kg/m^2c^ | 27.12 (26.86 to 27.38) | 27.12 (26.92 to 27.32) | 27.05 (26.73 to 27.37) | 0.9 |
| Smoking status, *n* (%) |  |  |  |  |
| Never | 226 (32.24) | 394 (34.87) | 131 (30.97) |  |
| Previous | 314 (44.79) | 452 (40) | 182 (43.03) |  |
| Current | 148 (21.11) | 271 (23.98) | 106 (25.06) | 0.2 |
| Alcohol, *n* (%) |  |  |  |  |
| <8 | 224 (31.95) | 365 (32.3) | 138 (32.62) |  |
| 8 to 15 | 140 (19.97) | 220 (19.47) | 76 (17.97) |  |
| 16 to 39 | 212 (30.24) | 330 (29.2) | 133 (31.44) |  |
| >40 | 125 (17.83) | 214 (18.94) | 76 (17.97) | 0.9 |
| Physical activity, *n* (%) |  |  |  |  |
| Inactive | 94 (13.41) | 186 (16.46) | 73 (17.26) |  |
| Moderately inactive | 220 (31.38) | 318 (28.14) | 113 (26.71) |  |
| Active | 379 (54.07) | 618 (54.69) | 235 (55.56) | 0.2 |
| Marital status, *n* (%) |  |  |  |  |
| Married/cohabitating | 502 (71.61) | 824 (72.92) | 297 (70.21) |  |
| Not married/cohabitating | 63 (8.99) | 100 (8.85) | 39 (9.22) | 0.9 |
| Educational attainment, *n* (%) |  |  |  |  |
| Primary/none | 274 (39.09) | 450 (39.82) | 186 (43.97) |  |
| Secondary | 224 (31.95) | 346 (30.62) | 121 (28.61) |  |
| Degree | 191 (27.25) | 309 (27.35) | 108 (25.53) | 0.5 |
| ^a^ BMI = body mass index. | | | | |
| ^b^ *P*-values are from analysis of variance models where characteristics are continuous and chi-square test where characteristics are categorical. | | | | |
| ^c^ Geometric means are presented with 95% confidence intervals. | | | | |
| ^d^ Numbers may not add to total due to missing values. | | | | |
|  | | | | |
