# Supplementary material for: The role of plasma microseminoprotein-beta in prostate cancer: an observational nested case–control and Mendelian randomization study in the European prospective investigation into cancer and nutrition
Source: Ann Oncol. 2019 Apr 8;30(6):983–9. doi: 10.1093/annonc/mdz121 (PMC6594452; doi:10.1093/annonc/mdz121)
Supplement: mdz121_Supplementary_Data [file mdz121_supplementary_data.zip › mdz121-Suppl_data/Supplementary Table S8.docx]

| **Supplementary Table S8.** Odds ratio of prostate cancer for a per unit increase in MSP (ng/ml) for IV estimates and MR results using inverse-variance method with and without adjustment for circulating concentrations of total PSA (ng/ml)^a^ | | |
| --- | --- | --- |
| Study | OR (95% CI) | |
| **MSP** |  |  |
| PRACTICAL for incident cancer^b^ | 0.96 (0.95 to 0.98) | |
| EPIC (Excluding PRACTICAL) for incident cancer^c^ | 0.97 (0.95 to 0.99) | |
|  |  |  |
| All pooled | 0.96 (0.95 to 0.97) | |
|  |  |  |
| **MSP (adjusted for PSA)** |  |  |
| PRACTICAL for incident cancer^b^ | 0.97 (0.96 to 0.98) | |
| EPIC (Excluding PRACTICAL) for incident cancer^c^ | 0.98 (0.95 to 0.99) | |
|  |  | |
| All pooled | 0.97 (0.96 to 0.98) | |
| ^a^ MSP = microseminoprotein-beta; IV = instrumental variable; MR = Mendelian randomization; PSA = prostate-specific antigen; OR = odds ratio; CI = confidence interval; PRACTICAL = Prostate Cancer Association Group to Investigate Cancer Associated Alterations in the Genome; EPIC = European Prospective Investigation into Cancer and Nutrition; BPC3 = Breast and Prostate Cancer Cohort Consortium. | | |
| ^b^ The PRACTICAL [5] consortium was established in September 2008 to gain insight into the genetic architecture and mechanisms of prostate cancer risk. | | |
| ^c^ EPIC estimates were calculated using genetic data available from EPIC participation in BPC3[6] and the OncoArray consortium[7]; there was no overlap for samples used to calculate these EPIC estimates and those used to calculate estimates within PRACTICAL. | | |
|  | | |
